# Longitudinal Study: Swine Inflammation and Necrosis Syndrome in Suckling and Weaned Piglets Is Associated with Tail Length and Integrity in Slaughter Pigs

**DOI:** 10.3390/ani16010056

**Published:** 2025-12-24

**Authors:** Karien Koenders-van Gog, Thomas Wijnands, Mirjam Lechner, Gerald Reiner

**Affiliations:** 1 Lintjeshof Veterinary Practice, LH Vet Group, 6031 RK Nederweert, The Netherlands; k.koenders@lintjeshof.com (K.K.-v.G.); t.wijnands@lintjeshof.com (T.W.); 2 UEG Hohenlohe, 91567 Herrieden, Germany; mirjam.lechner@web.de; 3 Clinic for Swine—Herd Health Management and Molecular Diagnostics, Justus-Liebig-University Giessen, 35392 Giessen, Germany

**Keywords:** animal welfare, tail damage, tail biting

## Abstract

Swine Inflammation and Necrosis Syndrome (SINS) is a highly prevalent, predominantly endogenous condition that compromises tissue integrity and animal welfare across different life stages in pigs. Increasing evidence suggests that early-life SINS lesions may predispose pigs to tail damage later in life; however, longitudinal data remain scarce. This study investigated the association between SINS-related clinical signs in suckling piglets and weaners and subsequent tail integrity during fattening and at slaughter. In a longitudinal study, 352 piglets from two Italian farms producing Parma ham were followed from the suckling phase to slaughter. Although SINS signs were generally mild, pigs affected during the weaner phase showed a 3.5-fold increased risk of developing short tails during fattening. Furthermore, the probability of reduced tail length at slaughter increased from 33.5% to 65.8% in pigs with a history of SINS. Early-life SINS was significantly associated with impaired tail integrity both at the onset of fattening and at slaughter. These new findings highlight endogenous inflammation and necrosis in early life as important yet underrecognized welfare risk factors and suggest that SINS can be utilised as a point of care and early preventive strategies may substantially improve tail integrity and welfare outcomes at slaughter.

## Simple Summary

Tail biting is a highly relevant welfare problem in pigs with undocked tails. This is the first longitudinal study that investigates the correlation between SINS (Swine Inflammation and Necrosis Syndrome) in early life of pigs (suckling piglets and weaned piglets)—and the tail length and tail integrity at the time of fattening and slaughter. In this case, tail biting occurred mainly at the end of the piglet rearing period and at the commencement of the finishing phase, resulting in a reduction in tail length and integrity which was measured at the time of slaughter. The study found a correlation between both SINS in early life and tail lesions later in life, and also with tail length and integrity at the time of slaughter. Several factors that predispose pigs for SINS are known, such as genetics and several housing, feeding and management factors. As this study found a correlation between SINS in early life and later tail issues, this raises the opportunity for prevention and early intervention, to reduce the chances of later welfare issues and therefore improving pig welfare.

## 1. Introduction

Tail lesions represent a major welfare and economic concern in pig production, particularly in the European Union where routine tail docking as a preventive measure is prohibited (Council Directive 2008/120/EC [1]). Affected pigs may experience pain and stress, and tail injuries are associated with reduced performance, compromised carcass quality, and increased labor and treatment costs [2,3]. Consequently, farms are required to implement effective management and housing strategies to prevent tail and ear injuries. Increasing societal and trade demands for higher welfare standards further underscore the need for demonstrable measures ensuring intact tails [4]. Preventive strategies often require investments in space allowance, environmental enrichment, climate control, feeding practices, monitoring systems, and personnel [5,6], with estimated additional costs ranging from €10–31 per pig [7].

Reported prevalences of tail biting and tail lesions vary widely across Europe, from below 1% in Italy and Denmark [8,9], 1–4% in Sweden and Norway [2,10,11,12], 20–30% in Ireland and Germany [13,14,15], 37% in Switzerland [16], 35–50% in Finland [17,18,19] up to more than 70% in Northern Ireland [20,21]. Comparisons between studies are challenging due to differences in study objectives, lesion definitions, scoring systems, and whether acute or healed lesions were assessed [22].

In addition to injuries caused by biting, tail and ear lesions may also result from endogenous inflammatory processes, described as swine inflammation and necrosis syndrome (SINS [23]). SINS affects multiple body regions including tails, ears, teats, coronary bands, heels, and claws, and occurs across all age groups, from newborn and suckling piglets to weaners, fatteners, and AI boars [24,25,26,27,28]. Reported prevalence is 30–40%, with the most severe manifestations in weaners [29]. Its inflammatory nature has been demonstrated using clinical, histopathological, biochemical, metabolomic, transcriptomic, genetic, and genomic approaches [24,26,30,31,32]. SINS is influenced by both genetic and environmental factors, making it a valuable animal-based measure for identifying and mitigating inflammation- and necrosis-related welfare problems [25,26,29].

It has been hypothesized that SINS in early life may predispose pigs to tail and ear lesions during the fattening period and at slaughter [23,25,26]. If confirmed, this would represent an important animal-based measure with the potential to improve tail integrity and welfare outcomes in pigs. However, longitudinal data linking SINS in suckling piglets or weaners to tail lesions later in life are scarce. Therefore, the objective of the present study was to investigate these associations in a longitudinal cohort followed from the suckling phase until slaughter.

## 2. Materials and Methods

### 2.1. Study Design

The study was conducted in two independent cooperatives operating within the same integration in Italy. Farms were selected from the Italian integration as part of the TAILSCAN consortium (EU-funded project), based on their willingness and suitability to participate. Key selection criteria included location, logistics, and the presence of pigs with undocked tails. Additionally, the two farms differed in certain parameters, allowing for insights into the impact of these differences on SINS scores and tail integrity.

**Figure 1:**
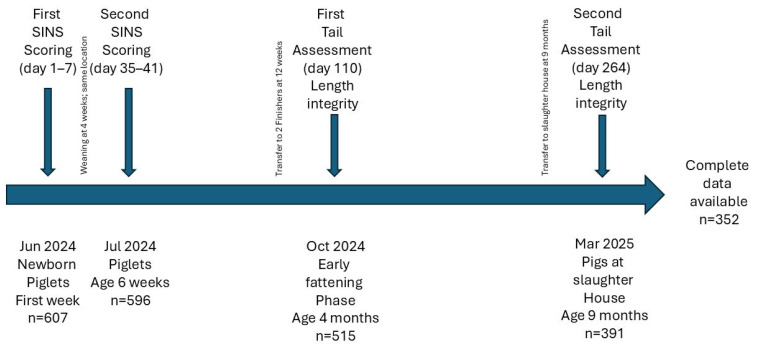
Layout of the experimental design.

The main differences between the farms were in health status, sow genetics, and nursery housing, whereas feed, boar genetics, and finishing systems were comparable. Each cooperative consisted of a sow herd, a nursery herd, and one or two finisher herds. Tail docking was not practiced. Piglets were weaned at four weeks of age, transferred to the finisher unit at twelve weeks, and slaughtered at an average age of 264 days. All units were part of Italian Parma ham production. Inflammation and necrosis (SINS) were recorded in suckling piglets and weaners at 1–7 days and 35–41 days of age, respectively. Tail integrity was assessed at the beginning of the fattening phase (110 days) and at slaughter (Figure 1).

Data were available for 566 suckling piglets and weaners, 473 fatteners, and 370 slaughter pigs. The study was conducted longitudinally, with suckling/weaning, fattening, and slaughter data collected for the same individuals. Complete data were available for 339 pigs, while the remaining animals could not be tracked to slaughter or their ear tags were missing.

### 2.2. Farm Characteristics

Sow Farm 1

Sow Farm 1 housed *n* = 750 sows of PIC genetics and used Fomeva (parity 1) and Goland (parity >1) boars for artificial insemination. The farm was located in an area of low swine density and was considered PRRS-stable, defined by long-term negative PCR results from processing fluids. Gilts were raised in a higher-density swine area and vaccinated against PRRS with a modified live vaccine. Piglets were not vaccinated against PRRS, but were vaccinated against PCV2 and *Mesomycoplasma hyopneumoniae*.

Lactating sows were housed in crates and fed ad libitum with dry meal feed supplied manually at least four times daily. Sows had nipple drinkers and received additional water via a trough with a tap every morning, with an average intake of 26 L/day. Piglets had nipple drinkers and additional water in bowls. Enrichment consisted of chains and nesting material made of paper. Climate conditions were favorable, with low CO_2_ levels (450–950 ppm) and incoming air cooled by a water system. Flooring in the farrowing unit consisted of plastic slats, with cast iron slats under the sow, and a piglet nest was located at the head side.

Gestating sows were housed in groups and had access to 13–17 L water/day via easily accessible drinkers. Feed contained no toxin binders, and mycotoxin levels were within acceptable limits. Feed was provided in two phases during gestation. During the first weeks, sows were housed in crates. Piglets were weaned at four weeks of age.

Nursery 1 was straw-bedded, housing 150 pigs per pen, with 10 nipple drinkers and 15 feeding places. Piglets were fed meal for the first two weeks post-weaning, then switched to pelleted feed. Nursery stay lasted until 12 weeks of age.

Sow Farm 2

Sow Farm 2 housed *n* = 900 sows using semen from Topigs, with Fomeva boar genetics. The farm operated on a three-week batch farrowing system. The health status was defined by the presence of a PRRS field strain infection, and clinical signs of arthritis were observed in suckling piglets. Piglets were vaccinated against PCV2 and *Mesomycoplasma hyopneumoniae*, but not PRRS.

The farm was situated in a higher swine density area than Farm 1. Gestating sows were housed in groups of 98 individuals, fed via feeding stations, and had 8–10 drinkers per group. Water intake was not measured. Enrichment included chains with wooden blocks and occasional straw/hay.

Lactating sows were housed in crates with plastic slatted floors and fed twice daily with pelleted feed via a valve-operated trough. Climate conditions were relatively warm (29 °C), with low CO_2_ levels. Sows and piglets had access to nipple drinkers. No nesting material or additional water sources were provided. Enrichment consisted of a movable tool attached to the crate.

Nursery 2 featured concrete flooring, 80 pigs per pen, 10 feeding places, 5 nipple drinkers, and 2 open water troughs per pen. Health issues at assessment included weaning diarrhea and coughing. Enrichment consisted of ropes.

#### Finishing Units

Finishers were housed on concrete flooring in pens of 20–40 pigs, fed liquid feed (including whey) three times per day with additional drinkers (1 per 10 pigs), and received rope enrichment. The farm operated under an antibiotic-free concept, with pigs only treated individually if necessary and marketed separately.

### 2.3. Animal Assessment

Pigs originating from the two Italian sow herds were individually monitored from the first week of life until slaughter. Animals were identified using digital ear tags (LeeO). Clinical assessments were conducted at three time points: during the first week of life, two weeks after weaning, and three weeks after transfer to the finishing unit. At slaughter, digital photographs of the tails were taken and evaluated.

**Figure 2:**
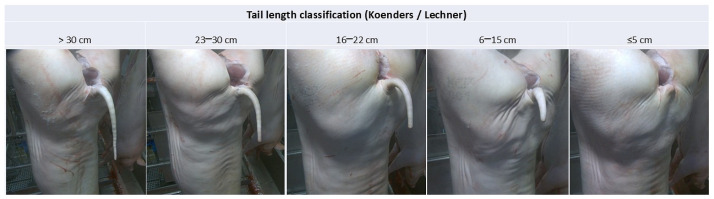
Tail length classification according to Koenders and Lechner.

**Figure 3:**
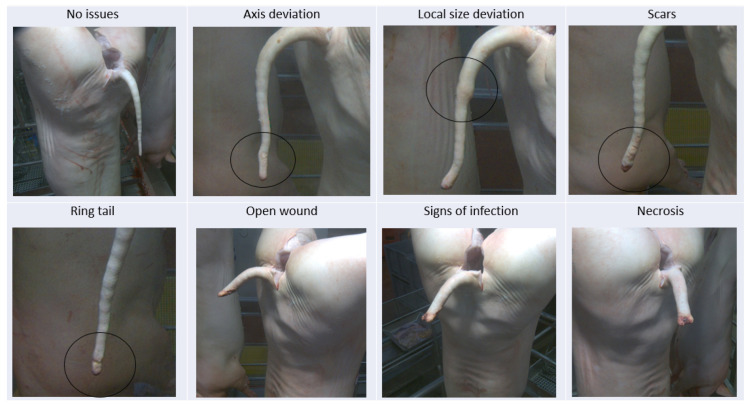
Tail lesion classification at slaughter according to Koenders and Lechner (see also Table 3). The circles in the figures highlight the respective changes.

All assessment procedures were non-invasive. Evaluations of fattening and slaughter pigs were performed by a single examiner, whereas assessments of suckling piglets and weaners were carried out by two examiners, who worked in close coordination and strictly adhered to standardized assessment protocols (see Table 1 and Table 2; Figure 2 and Figure 3). This approach ensured a high level of consistency and reproducibility across all assessment time points, and examiner-related bias was therefore considered unlikely.

The study was conducted in a partially blinded manner, as results from other time points were not available to the examiners during each assessment. At the slaughterhouse, examiners were additionally blinded to the farm cooperative of origin, further minimizing potential sources of bias.

The following clinical parameters were assessed in neonatal and weaned pigs and recorded as present or absent (see Table 1).

**Table 1:** Neonatal and weaned pigs clinical assessment.

Tail base	Hairless	Less than normal or no hair visible
	Redness	Redness of the skin is visible
	Swelling	Palpable subcutaneous oedema
	Exudate	Inflammatory liquid visible at the surface
	Necrosis	Necrotic tissue visible at the surface
	Bleeding	Blood is visible
Tail tip	Redness	Redness of the skin is visible
	Swelling	Palpable subcutaneous oedema
	Exudate	Inflammatory liquid is visible at the surface
	Necrosis	Necrotic tissue is visible at the surface
	Bleeding	Blood is visible
Ear basis	Hairless	Less than normal or no hair is visible
	Redness	Redness of the skin is visible
	Exudate	Inflammatory liquid visible at the surface
Ear tip	Redness	Redness of the skin is visible
Coronary band	Redness	Redness of the skin is visible
	Exudate	Inflammatory liquid is visible at the surface
	Swelling	Swollen tissue is visible
	Subcutaneous bleeding	Blood is visible
Teats	Redness	Redness of the teats is visible
	Swelling	Swollen tissue is visible
	Necrosis	Necrotic teats (black, dry) are visible

After weaning, additionally, ear wounds were noted. At 3 weeks post placement in the finishing units, only the pigs’ tails were assessed according to the following scheme (Table 2):

**Table 2:** Finishing pigs’ tail assessment.

Score 1	tail long and intact, with curl, no lesions
Score 2	tail long and intact, but shorter, still with curl, no lesions
Score 3	tail is shorter, no curl, healed, no acute lesions
Score 4	tail long with acute lesion bite wound
Score 5	tail is shorter, with acute lesion bite wound

### 2.4. Tail Assessment at the Slaughterhouse

Pigs were slaughtered at a minimum age of 9 months, in accordance with the requirements of Parma ham production. At slaughter, three digital photographs of each individual pig’s tail were obtained and visually assessed using a TAILSCAN camera system (Farm4Trade, Abruzzo, Italy). Photographs were taken from a standardized angle using a camera mounted on a robotic arm. Tail length was measured on one of the three photographs and classified into five categories: >30 cm, 23–30 cm, 16–22 cm, 6–15 cm, and <5 cm, as illustrated in Figure 2. In addition, pigs were dichotomized into long-tailed (>23 cm) and short-tailed (<23 cm) groups. This classification was applied because the two groups exhibited clearly opposing patterns in their associations with the investigated characteristics.

Tail integrity was evaluated by visual assessment of all 3 photos taken from 3 different angles of the same tail, according to the scheme in Table 3. This is illustrated by Figure 3.

**Table 3:** Tail integrity in slaughter pigs.

Tail Lesion Categorization	Description
No issues	
Knick/Axis deviation	A deviation from the normal longitudinal shape of the tail: sideways deviation
Local size deviation	A deviation from the normal circumference of the tail: local thickening
Scars	Visible signs of healing process of the surface of the tail: uneven surface
Ring tail	A local constriction of the tail circumference
Open wound	Visible signs of lack of skin surface integrity
Signs of infection	Visible signs of (chronic) inflammatory reaction of the body such as swollen tissue and granulomatous tissue
Necrosis	Visible signs of dead tissue (discoloration, dry and hard tissue)

### 2.5. Statistical Analysis

Individual combined data of suckling piglets and weaners were available from 566 piglets. Data from the fattening phase was available from 515 fatteners. Slaughter data were available from 370 pigs. Combined piglet and fattening data were available from 494 pigs. Combined fattening and slaughter data were available from 363 pigs. 352 pigs had the full information with data from the suckling piglet phase until slaughter. Three farm coops were involved with two sow/nursing/fattener combinations and one slaughterhouse. Farm coop 1 had 50.4% of the suckling piglets to fatteners and 57.4% of the slaughter pigs. Farm coop 2 had 49.6% of the suckling piglets to fatteners. Most losses occurred because of logistical problems and the loss of ear tags.

All data were collected and organized in Microsoft Excel (Microsoft, Munich, Germany) prior to statistical analysis using IBM SPSS Statistics version 29 (IBM, Munich, Germany). The prevalence of the examined clinical signs in the various body parts was calculated from binary observations (procedure crosstabs). For each body part, the sum of all binary variables (e.g., absence of bristles [0/1], redness [0/1], swelling [0/1], exudation [0/1], necrosis [0/1], bleeding [0/1]) was used to derive a body-part-specific score (e.g., tail base score). An overall SINS score was then obtained by summing all body-part scores. Associations were analyzed using the procedure generalized linear mixed models (GLMMs), which allow for normally and non-normally distributed outcomes, account for the hierarchical structure of the data (piglets nested within sows, sows nested within farms), and accommodate repeated measurements and multiple fixed effects, including biologically relevant interactions. Piglet identity was included as a random effect, and day of life was treated as a repeated measure. Sex, parity, and farm cooperative were included simultaneously as fixed effects, while sow nested within farm was included as a random effect to account for the hierarchical data structure. Biologically plausible two-way interactions between SINS variables and fixed effects (e.g., farm × SINS, age × SINS) were explored. Interaction terms were retained in the final model only if they were statistically significant or improved model fit. Binary outcome variables were analyzed using a binomial distribution with a logit link function, whereas continuous outcome variables were analyzed using linear mixed-effects models.

To evaluate the association between individual SINS-related clinical signs and outcome variables, multivariable mixed-effects models were fitted, including one SINS variable at a time as a fixed effect while adjusting for all relevant covariates. Again, biologically plausible two-way interactions between SINS variables and fixed effects (e.g., farm × SINS, age × SINS) were explored. Interaction terms were retained in the final model only if they were statistically significant or improved model fit. This approach was chosen because including multiple correlated binary SINS variables as fixed effects together with the biologically essential random sow effect led to convergence issues. Thus, a conservative modeling strategy was adopted to ensure robust and interpretable estimates.

Associations between fattening data, piglet data, and slaughter data were analyzed using Chi-square tests (implemented in crosstabs). Figures show means ± standard errors. Values with the same superscript letter were not significantly different. Statistical significance was defined as *p* < 0.05. Individuals with missing date were excluded from analysis.

## 3. Results

### 3.1. SINS Signs in Suckling Piglets and Weaners

Suckling piglets and weaners exhibited only minor SINS-related lesions (Table 4 and Table 5), with five characteristics undetectable in piglets and fifteen in weaners. Lesions observed included exudation, necrosis, and bleeding at the tail base and tip, exudation at the ears, and teat necrosis, mostly at low prevalence. No SINS manifestations were detected in the coronary bands, heels, or teats of weaners.

Coronary band exudation was observed in 58% of suckling piglets and was considered an outlier, as the prevalence of all other SINS signs averaged 2.8% (range: 0–16.1%). Weaners showed a similar pattern, with 1.6% affected (range: 0–20.5%). On average, individual body parts were affected in 18.9% of suckling piglets, with a maximum prevalence of 61.8%, whereas in weaners, the mean prevalence was 5.3% (maximum 21.8%).

Overall, the prevalence of SINS at the individual animal level was 72% in suckling piglets and 29% in weaners (Table 5).

The SINS scores of suckling piglets ranged from 0.04 (tail tip) to 0.63 (coronary bands) and for weaners from 0 (teats) to 0.24 (tail base) (Table 6), reflecting low overall severity but clear distribution patterns influenced by farm, sex, age, and sow parity. Farm had a substantial effect on tail base scores in piglets and ear and teat scores in weaners (Table 6). Sex significantly affected teat scores in piglets and ear scores in weaners (Table 6). Age influenced tail base and heel scores, and parity affected multiple body parts (Table S1). First parity piglets generally had the lowest SINS scores, which increased until the fourth parity, whereas weaners of the third and fifth parity were more severely affected than those of the first and fourth parity. (Table S2).

**Figure 4:**
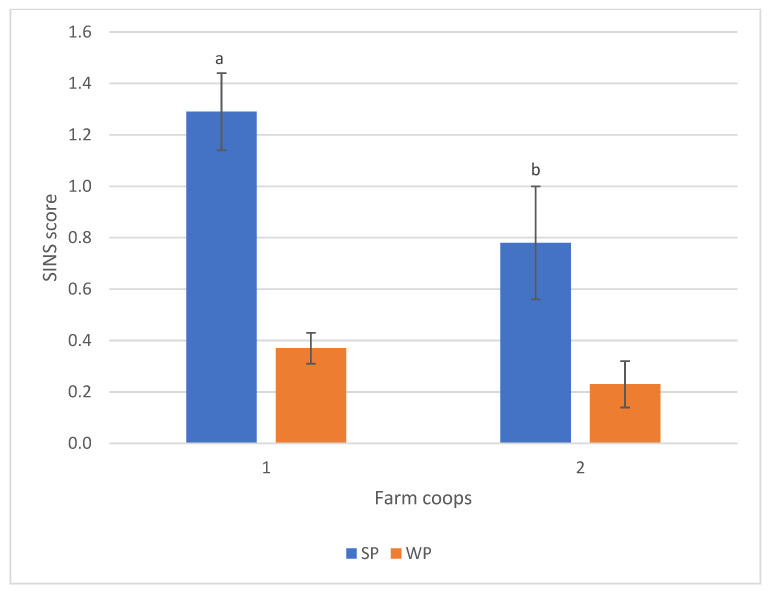
SINS scores of suckling piglets (SP) and weaners (WP) at the two farm combinations. Numbers: *n* = 285 (farm coop 1); *n* = 280 (farm coop 2). Bars show mean values; whiskers show standard errors. Bars within one category with different letters differ statistically significantly (*p* ≤ 0.05).

When body part scores were combined into a total SINS score, the average was 0.93 ± 0.16 for suckling piglets and 0.29 ± 0.06 for weaners. The combination of farrowing and nursery farms significantly influenced the SINS score in suckling piglets (Figure 4).

**Figure 5:**
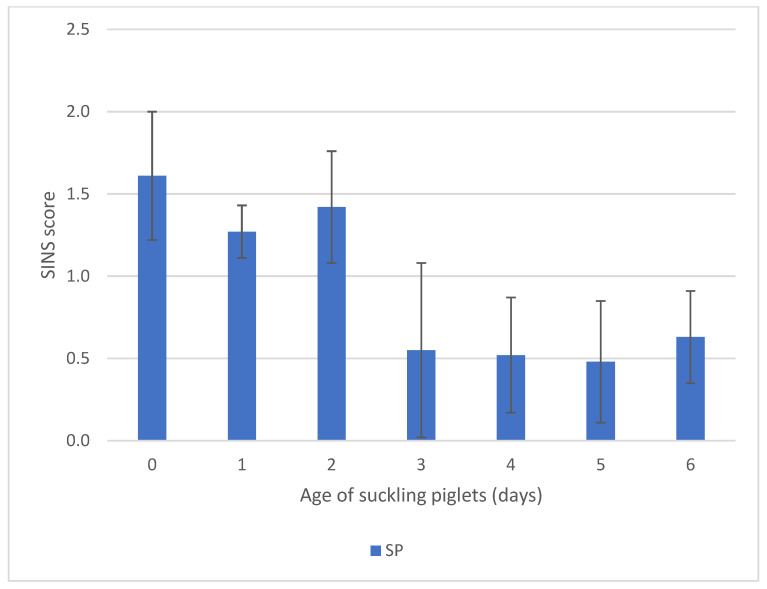
SINS scores of suckling piglets (SP) and weaners (WP) by piglets age (days). For numbers of cases see Table S1. Bars show mean values; whiskers show standard errors.

SINS scores in suckling piglets tended to be higher from birth to day 2 compared to days 3 to 6. However, given the overall low scores and marked variability, differences between days did not reach statistical significance (Figure 5).

**Figure 6:**
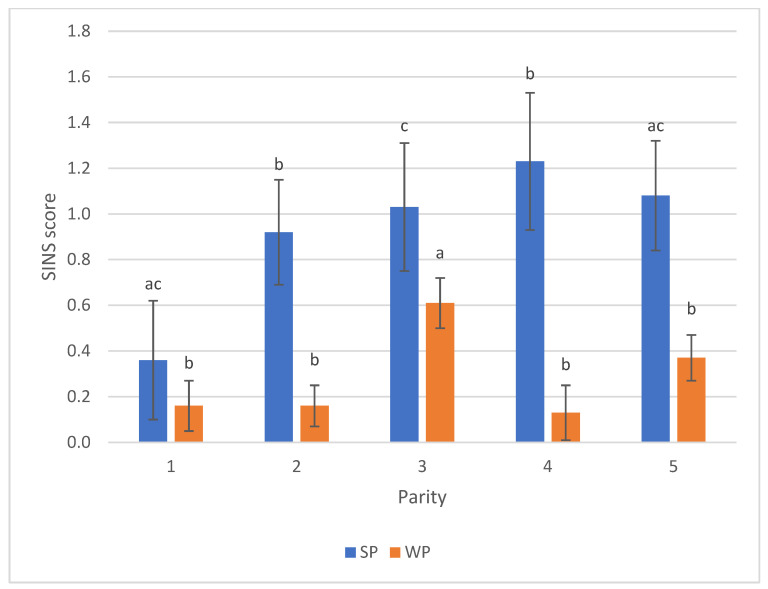
SINS scores of suckling piglets (SP) and weaners (WP) by parity of the sow. For numbers of cases see Table S2. Bars show mean values; whiskers show standard errors. Bars within one category with different letters differ statistically significantly (*p* ≤ 0.05).

Sow parity had a pronounced influence on SINS scores in suckling piglets. Scores initially decreased in the first parity but subsequently increased, peaking around the fourth parity. The third parity displayed greater uniformity and differed significantly from all other parities. A significant difference was also observed in the SINS scores of weaners from the third parity compared to other groups (Figure 6). Conversely, sex did not notably influence SINS scores in either suckling or weaned piglets; values differed only slightly for suckling piglets (0.88 males vs. 0.97 females) and were virtually identical for weaners (0.28 males vs. 0.29 females).

### 3.2. Tail Scores at Early Fattening Phase

At the start of the fattening phase, 45% of the pigs had long, intact tails, 8% had short tails without lesions, 8% had short tails with healed lesions, 29% had long tails with acute lesions, and 11% had short tails with acute lesions (Table 7). The acute lesions resulted from biting injuries that originated at the end of the nursery phase. By finisher 3, pigs showed a marked reduction in the incidence of acute tail lesions, whereas the prevalence of short tails—both with and without healed lesions—increased significantly. In this study, neither sow parity nor pig sex had a significant effect on tail scores during the fattening period.

### 3.3. Association Between SINS in Suckling Piglets and Weaners and Tail Score During Fattening Phase

**Figure 7:**
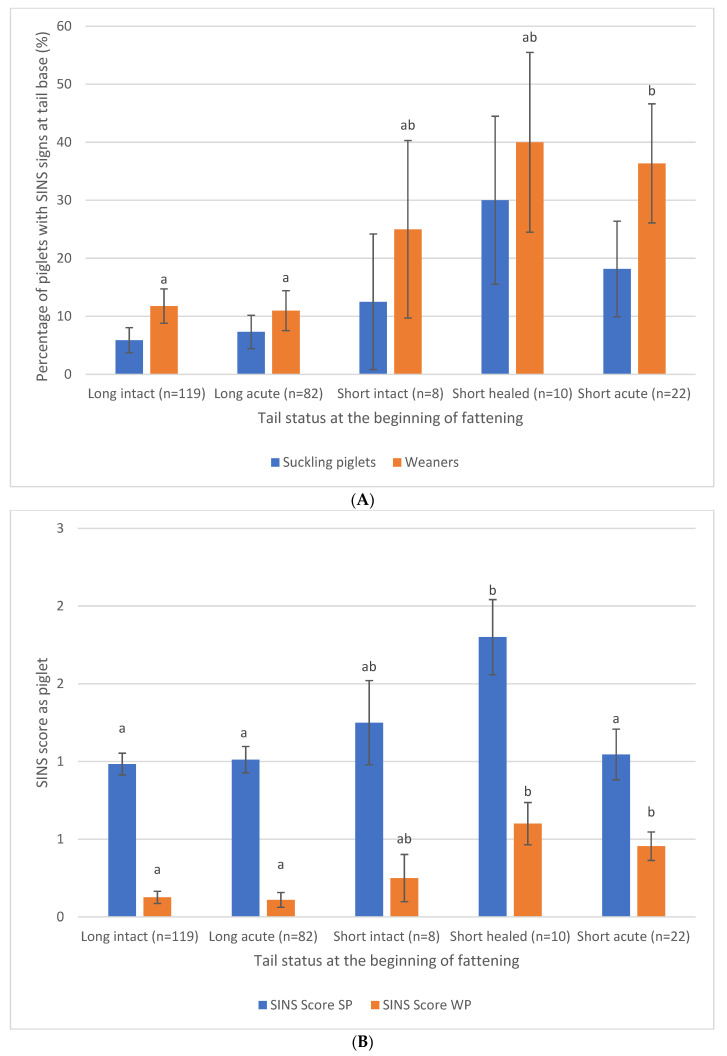
(**A**) Percentage of suckling piglets (blue columns) and weaners (orange columns) with SINS signs at the tail base, as associated with their tail status at the beginning of the fattener phase in farm coop 1. Bars show mean values; whiskers show standard errors. Bars within one category with different letters differ statistically significantly (*p* ≤ 0.05). (**B**) SINS score in suckling piglets (blue columns) and weaners (orange columns) as associated with their tail status at the beginning of the fattener phase in farm coop 1. Bars show mean values; whiskers show standard errors. Bars within one category with different letters differ statistically significantly (*p* ≤ 0.05).

**Figure 8:**
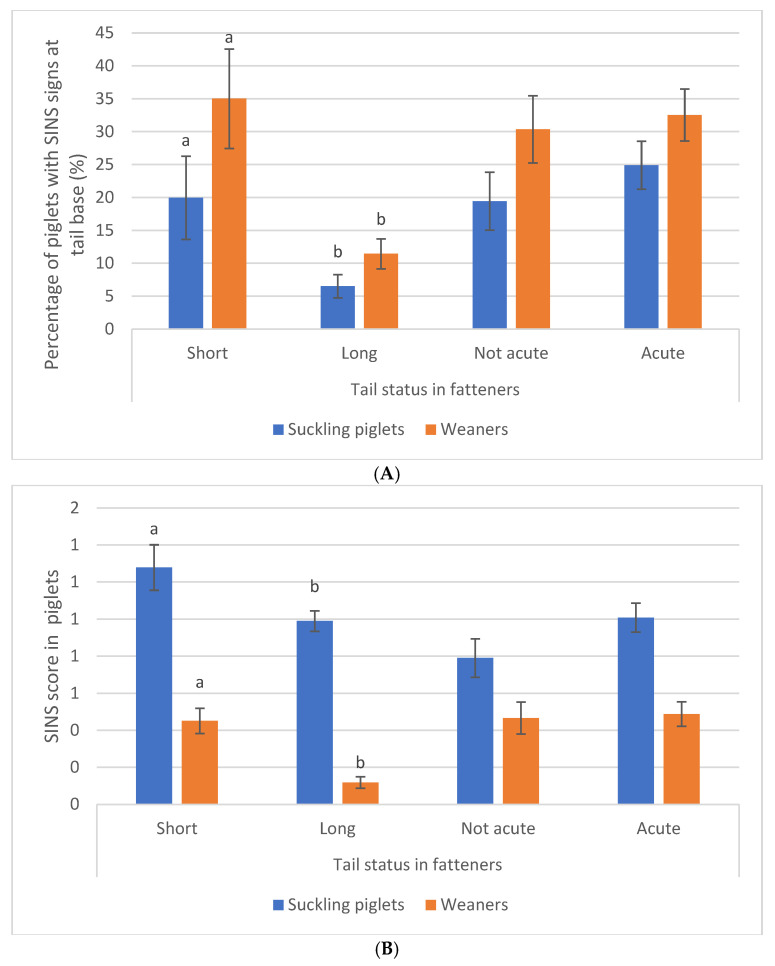
(**A**) Percentage of suckling piglets (blue columns) and weaners (orange columns) with SINS signs at the tail base, as associated with their tail length and acuteness of their tail lesions at the beginning of the fattening phase in farm coop 1. Bars show mean values; whiskers show standard errors. Bars within one category with different letters differ statistically significantly (*p* ≤ 0.05). (**B**) SINS score in suckling piglets (blue columns) and weaners (orange columns) as associated with tail length (>23 cm vs. <23 cm) and acuteness of tail lesion at the beginning of the fattener phase in farm coop 1. Bars show mean values; whiskers show standard errors. Bars within one category with different letters differ statistically significantly (*p* ≤ 0.05).

Fatteners with shorter tails were more likely to have exhibited SINS signs at the tail base (Figure 7A) and SINS overall (Figure 7B) as suckling piglets (blue bars) and, even more so, as weaners (orange bars). In summary, SINS status during the piglet phase was associated with tail length at the fattening stage (Figure 8A,B), but not with the acuteness of the tail lesions in fatteners.

**Figure 9:**
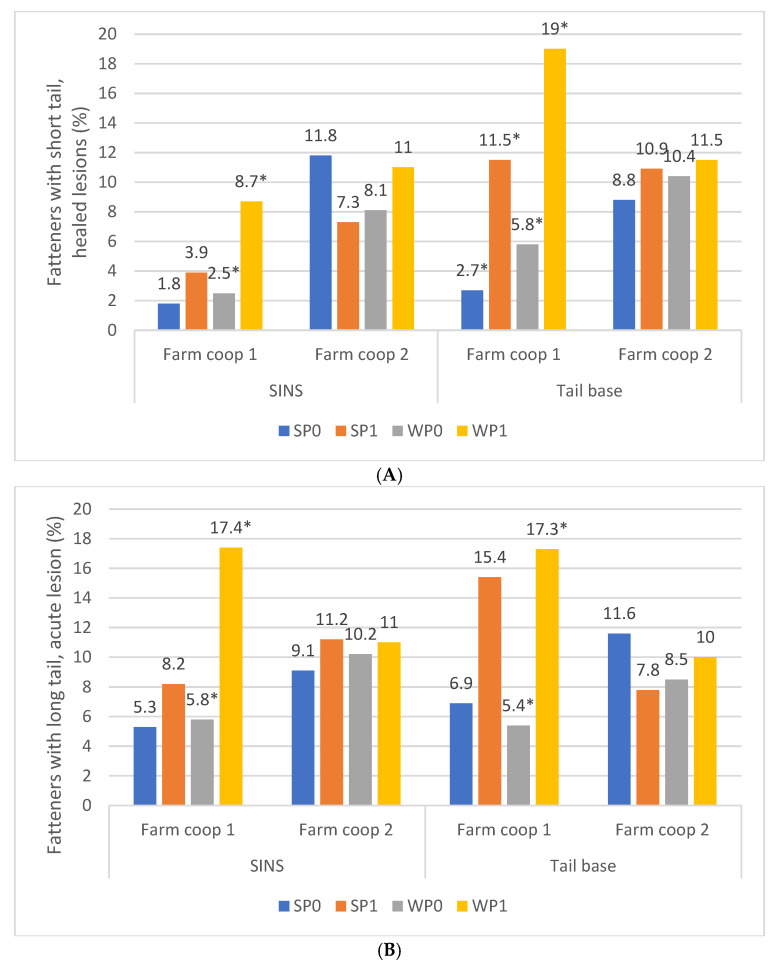
(**A**) Percentage of fatteners exhibiting shorter tails and healed lesions, according to their status of tail base and SINS as suckling piglets and weaners, by farm coop. SP0: suckling piglets without signs of SINS (farm coop 1/farm coop 2: *n* = 275/*n* = 254; SP1: suckling piglets with signs of SINS (*n* = 10/*n* = 26); WP0: weaners without signs of SINS (*n* = 279/*n* = 262); WP1: weaners with signs of SINS (*n* = 22/*n* = 30). Bars show percentage of piglets; *: values within one farm coop are significantly different at *p* ≤ 0.05. (**B**) Percentage of fatteners exhibiting longer tails and acute lesions, according to their status of tail base and SINS as suckling piglets and weaners, by farm coop. SP0: suckling piglets without signs of SINS (farm coop 1/farm coop 2: *n* = 267/*n* = 258; SP1: suckling piglets with signs of SINS (*n* = 22/*n* = 30); WP0: weaners without signs of SINS (*n* = 263/*n* = 250); WP1: weaners with signs of SINS (*n* = 22/*n* = 30). Bars show percentage of piglets; *: values within one farm coop are significantly different at *p* ≤ 0.05.

Fatteners showed a higher propensity for shorter, healed tails if they had displayed SINS signs during the suckling and weaning phases on farm cooperative 1, whereas this pattern was not observed on farm cooperative 2 (Figure 9A,B). On farm coop 2, SINS signs were generally elevated, and pigs—whether affected or unaffected—showed similar rates of shorter tails with healed lesions during fattening. In contrast, on farm coop 1, the presence of SINS signs during the suckling period—and to a greater extent during the weaning period—was significantly correlated with the development of shorter, healed tails during fattening. Specifically, SINS signs at the tail base were associated with a three- to fourfold increase in the likelihood of developing shorter healed tails in fatteners.

A similar outcome was observed when analyzing fatteners with long tails that exhibited acute lesions. Weaners from farm coop 1 showing signs of SINS had a threefold increased probability of developing acute lesions during the fattening phase compared to weaners without such signs (Figure 8B).

### 3.4. Tail Scores at Slaughter

Following the biting insult at the end of the nursery phase, a large proportion of slaughter pigs exhibited healed scars (73%), while 25% showed no adverse effects. The combination of nursery 1 and finishing at fattener 1 imposed a lower burden on the tails than the combination of nursery 2 and finishers 2 and 3, as pigs in the former group had significantly fewer tail issues and scars. The prevalence of kinks, size deviations, ring formation, wounds, signs of infection, and necrosis in slaughter pigs ranged from 3.3 to 6.5% (Table 8). No obvious effects of piglet sex or sow parity were observed. Significant differences in the prevalence of pigs without issues and pigs with healed scars were noted between farms, with the combination of farrowing farm 1, nursery farm 1, and finisher farm 1 yielding optimal outcomes, whereas the combination of farrowing farm 2, nursery farm 2, and finisher farm 2 was suboptimal.

The tail length at slaughter correlated directly with the issues observed post-mortem. The farm cooperative with the fewest issues had the highest proportion of pigs with long tails (≥23 cm), while the farm cooperative with the most issues had the majority of pigs with short tails (<23 cm) at slaughter (Table 9). The long/short tail ratios were 78.2%/22.8% for the farm with the fewest issues and 23.5%/76.5% for the farm with the most issues. Extremely short tails (<15 cm) were relatively rare. Again, no effects of parity or sex were detected.

Beyond kinks, tail condition during fattening was strongly associated with tail condition at slaughter (Table 10). Tails exceeding 30 cm at slaughter were frequently observed, especially in pigs that remained intact throughout the fattening phase. Long tails (≥23 cm) were significantly less common in pigs that already had shorter tails with healed or acute lesions at the beginning of fattening. Additionally, healed scars appeared in pigs that had shown no lesions during fattening, and the prevalence of these lesions increased significantly in pigs with acute lesions at the start of fattening. Ring constrictions were rare, occurring mainly in pigs with shorter tails that had already healed lesions during fattening.

### 3.5. Association Between SINS Signs in Suckling Piglets and Weaners and Tail Scores at Slaughter

**Figure 10:**
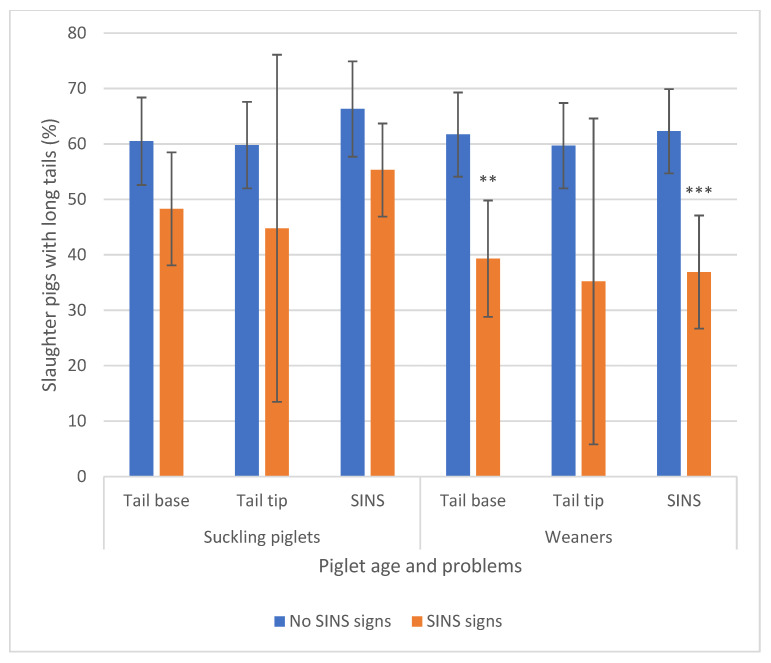
Prevalence of slaughter pigs with long tails (>23 cm) in association with signs of SINS at the tail base, tail tip and with the SINS score of these pigs as suckling piglets and weaners. These results are based on 352 pigs. Bars show mean values; whiskers show standard errors. Values are significantly different: ** *p* < 0.01; ***: *p* < 0.001.

Pigs without any SINS signs, either overall or at the tail base or tail tip, as suckling piglets or weaners (Figure 10, blue bars), exhibited long tails (≥23 cm) in approximately 60% of cases. This prevalence decreased markedly when pigs displayed SINS signs as suckling piglets and, particularly, as weaners. However, due to high variability, these effects were only statistically significant for SINS and tail-base signs in weaners.

**Figure 11:**
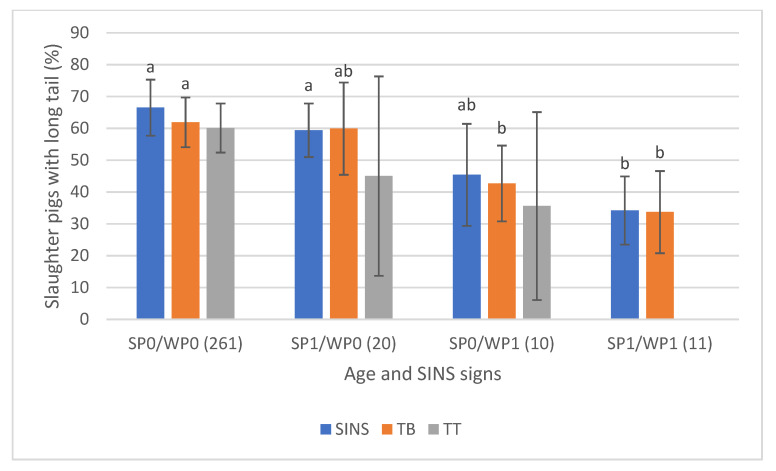
Prevalence of slaughter pigs with long tails (>23 cm) in association with signs of SINS at the tail base (TB), tail tip (TT) and with the SINS score of these pigs as combination from suckling piglet (SP) and weaner (WP) results. SP0/WP0: these pigs had neither signs of SINS as suckling piglets nor as weaners. Numbers of piglets in brackets). Bars show mean values; whiskers show standard errors. Bars within one feature with different letters differ statistically significantly (*p* ≤ 0.05).

The association was particularly evident when examining SINS signs at both the tail base and tail tip (Figure 11). Approximately 60% of pigs with no SINS indications as suckling piglets (SP0) and weaners (WP0) had long tails (average ≥23 cm). This prevalence dropped significantly to below 40% for pigs affected by SINS at both stages (SP1/WP1). Notably, some pigs had unaffected distal tail regions at both ages. Overall, changes occurring during the weaning stage were more strongly associated with tail findings at slaughter than those occurring only during the suckling phase.

When tail outcomes in the fattening phase were split into length and acuteness components (Table 11), longer tails at slaughter had been longer throughout fattening. Pigs with minimally shorter tails during fattening rarely developed tails >30 cm at slaughter. Longer tails during fattening were significantly less likely to be <23 cm at slaughter and were associated with fewer healed scars and ring constrictions. Pigs with acute lesions during fattening had significantly shorter tails at slaughter and more healed scars.

## 4. Discussion

### 4.1. SINS Prevalence and Scores

In this longitudinal study, the prevalence of inflammation and/or necrosis (SINS) across examined body parts was surprisingly low. The average prevalence per SINS characteristic and body part was 1.7% and 1.41% in suckling piglets and weaners, respectively. The tail base and tip were affected in approximately 3% of suckling piglets, while the ear base showed lesions in up to 4%. Comparable prevalences were observed in weaners, with 7.4% exhibiting redness at the tail base. All other body parts showed even lower prevalence rates. These findings contrast sharply with previous studies reporting much higher prevalences, including 39% for the tail base, 36.4% for the tail tip, 36.2% for the ears, 32% for teats, 45.8% for coronary bands, and 72.9% for heels [29]. Interestingly, exudation of the coronary bands affected 64% of suckling piglets in the present study. This marked discrepancy suggests that additional mechanical or environmental factors may have contributed, although these could not be clearly identified.

Higher-grade SINS signs, such as exudation and necrosis, occurred at frequencies comparable to previous studies [24,25,26,27,28]. Lower rates of teat necrosis were observed in both suckling piglets and weaners compared with prior reports, yet the increased vulnerability of female piglets to SINS manifestations at the teats was corroborated [25,29].

The low prevalence of SINS in the two Italian herds studied may be linked to specific production parameters of Parma ham [33]. This system is characterized by slower growth, higher fat deposition, specific genetics, and regulated slaughter age and weight. Parma ham pigs are slaughtered at a minimum of 9 months of age and ≥160 kg live weight, and the ADG in the final fattening phase is approximately 720 g/day, substantially lower than in Germany (up to 1233 g/day) and Denmark (1039 g/day) [34,35,36,37]. Feed conversion ratios are higher (3.7 kg feed/kg gain vs. 2.44–2.67 in Germany and Denmark) and feed is tailored for gradual growth to achieve the desired ham quality. Carcass fat composition targets a subcutaneous fat layer of 20–30 mm, markedly higher than 16 mm in the Netherlands, with lean meat percentages in Germany and Denmark around 60% [34,35,36,37].

Previous research indicates that higher SINS scores are associated with genotypes selected for rapid growth and high meat yield [28,31]. Consequently, the slower growth, higher fat deposition, and controlled genetics in Parma ham production likely contribute to the lower SINS scores observed. The feed composition may also play a critical role, as diets excessively rich in protein and starch but low in fiber can overload intestinal and hepatic metabolism, triggering systemic inflammation, including SINS [23].

### 4.2. Tail Length and Integrity

Tail lesions were influenced by Italian regulations banning routine tail docking, leading to a high proportion of undocked pigs. At slaughter, 25.3% of pigs had intact tails, 72.8% showed healed lesions, and 3.3% displayed fresh wounds or necrosis. Ringtails were observed in 4.2% of pigs, a phenomenon previously linked to inflammation in pigs [23], cattle [38], and rodents [39]. The relatively high prevalence of tail lesions in this study is likely due to meticulous longitudinal assessments, the ASF-related temporary overcrowding event in the region, and the limited sample size. At the point of slaughter, the examination was done based on digital images of the tails. During this examination, even minor deviations were meticulously documented as findings. It is evident that such subtle variations cannot be reliably detected under practical conditions in larger slaughterhouses, particularly when the primary focus is on meat inspection. A notable shortcoming of the study is the well-known lack of methodological consistency between the different studies on tail lesions, which is also apparent in the present study [22]. Furthermore, it is well documented that slaughter findings are less suitable for characterising tail lesions than direct examination of live animals in the barn [15,18,21,40].

Significant associations between early-life SINS and tail lesions were evident. Weaners with SINS had a 3.48-fold higher probability of short tails with healed lesions during fattening, whereas suckling piglets exhibiting SINS at the tail base had a 4.3-fold increased risk of shorter tails and healed scars. Even mild SINS manifestations can thus serve as animal-based measures (ABMs) for predicting tail integrity at slaughter, in line with previous research [19].

### 4.3. Farm Effects, Management Factors and Practical Implications

The pronounced farm effects documented for French [29] and Dutch [25] farms were also observed in the present study. Farm 1 demonstrated lower tail lesion prevalence and stronger associations between early SINS and tail outcomes, likely attributable to improved nursery conditions, including straw bedding, higher sow health status (e.g., PRRS stability), and reduced stress, which are known to influence the level of SINS [23]. Conversely, farm 2 exhibited higher tail lesion prevalence, potentially due to ASF-related overcrowding, less favorable nursery conditions (concrete floors), and health challenges such as post-weaning diarrhea and respiratory disease.

On farm 1, tail biting may therefore have been a selective phenomenon, with attacks primarily directed at animals that had already sustained damage and were therefore perceived as being more attractive. As demonstrated in a substantial body of research, animals that have sustained pre-damage are susceptible to an increased incidence of biting injuries [41,42,43,44,45,46]. Conversely, on farm 2, the role of attraction may have been less significant due to elevated stress levels with a reduced threshold, resulting in a more widespread manifestation that was detrimental to associations. This is also supported by the fact that the damage to coop 2 occurred with less prior signs of inflammation and necrosis compared to coop 1. Differences in sow genetics, feeding management, and feed composition may have further contributed to observed disparities [23].

Under relatively low-stress conditions in farm 1, associations between SINS scores in suckling piglets and weaners and tail lesions during fattening and at slaughter were clearly evident. These results demonstrate that even mild to moderate SINS manifestations can serve as early predictors of tail integrity and provide opportunities for proactive herd intervention. Further, these findings underscore the importance of early-life management, including proper gestating sow care (adequate water supply, enrichment, prevention of coprostasis), optimal suckling piglet management, and attention to the weaning phase, the most stressful period in early pig life and the phase most strongly associated with observed outcomes [23,25,29].

The unique production parameters of Parma ham, including slower growth, higher fat content, and controlled genetics, appear to contribute to lower SINS prevalence and milder manifestations, suggesting that tailored early-life management and genetic strategies can effectively reduce welfare problems associated with inflammation and necrosis.

Taken together, the use of Swine Inflammation and Necrosis Syndrome (SINS) as an early point of care represents a promising preventive approach to reduce tail lesions in fattening and slaughter pigs and, consequently, to improve animal welfare. The relevance of tail lesions as a major welfare concern is well documented. Tail lesions are associated with acute stress and pain responses as well as with indications of long-term impairment, thereby exerting both immediate and chronic negative effects on animal welfare [2,5,47,48,49,50]. Importantly, pain may be present even in the absence of externally visible wounds [51]. Moreover, tail lesions can give rise to secondary infections and systemic complications, including abscess formation, osteomyelitis, and pyaemia [52]. The findings of the present study emphasize the importance of early-life management, genetics, and production system parameters in mitigating SINS and enhancing animal welfare. Integration of SINS monitoring as an animal-based measure in routine herd management represents a practical approach to reducing tail biting and improving pig health and welfare across the production lifespan.

Weaners with SINS exhibited a 3.48-fold increased probability of exhibiting short tails with healed lesions as fatteners. When considering the tail base alone, the chance was increased by 3.2. Furthermore, it was observed that suckling piglets exhibiting SINS signs at the base of the tail had a 4.3 higher chance to develop shorter tails and healed scars during fattening. Concurrently, the probability of developing acute lesions on the long tails of fattening pigs increased threefold if the pigs exhibited indications of SINS at the tail base or a SINS score during their weaning phase.

The presence of SINS signs at the tail base or a SINS score in weaners was associated with a 1.6-fold elevated probability of tail length < 23 centimetres in slaughter pigs. The results also suggest that inflammation and necrosis, even in mild to moderate forms, can be used as ABMs due to existing associations with important target characteristics, such as the integrity of the tail in slaughter pigs. This finding aligns with the conclusions of Becker et al. [28], who demonstrated that even minor variations in SINS scores of AI boars were associated with the SINS scores of their offspring. This finding is of particular importance because SINS signs occur early and can therefore be used for direct intervention in the affected herd [25].

Several management factors that are underlying SINS are known, such as: the availability of enrichment material and an optimal water supply, starting already in gestating sows [53]. It has been demonstrated that coprostasis in sows has a long-lasting negative effect on SINS in the offspring [25,53].

The implication of the correlation between SINS in early life and the tail length and integrity later in life—that is demonstrated in this study—is of big practical importance for swine husbandry and welfare, because it raises the opportunity to improve these factors in early life, to reduce the risk for welfare problems in later life of the pig.

## 5. Conclusions

The presented results provide initial evidence that endogenous inflammation and necrosis (SINS) may promote the development of tail lesions during fattening and at slaughter. This is the first longitudinal study to investigate the association between SINS and tail lesions with individual follow-up from birth until slaughter, representing a novel finding that confirms earlier indications of a link between SINS and tail biting.

Our findings highlight the importance of early-life management across the entire lifespan of pigs. Interventions should not be limited to the nursery or finishing phases, but should begin much earlier, including management of the pregnant sow (e.g., water intake, environmental enrichment), suckling piglets, and especially during the weaning phase, which is the most stressful and most strongly associated with our observed outcomes.

Strategies aimed at reducing the occurrence of SINS can be implemented and evaluated to determine their effect on decreasing tail biting, ultimately improving pig welfare. Future studies in larger cohorts and under varying housing conditions are warranted to confirm these findings and to optimize preventive management across all stages of pig production.

## Figures and Tables

**Table 4:** Percentage of piglets with signs of SINS by body part in suckling piglets and weaners (descriptive data).

		Suckling Piglets	Weaners
Tail base	No bristles	15.0	20.5
	Redness	1.3	4.9
	Swelling	1.3	1.2
	Exudation	0.5	0.3
	Necrosis	0.0	0.2
	Bleeding	0.0	0.3
Tail tip	No bristles	0.3	0.0
	Redness	1.3	1.0
	Swelling	0.5	0.0
	Exudation	0.8	0.0
	Necrosis	0.5	0.2
	Bleeding	0.0	0.2
Ear base	No bristles	0.5	0.0
	Redness	1.0	0.0
	Exudation	1.2	8.4
Ear tip	Redness	0.5	0.0
Teats	Redness	4.9	0.0
	Swelling	0.7	0.0
	Necrosis	7.9	0.2
Coronary bands	Redness	4.4	0.0
	Exudation	58.0	0.0
Heels	Swelling	1.8	0.0
	Redness	16.1	0.0

**Table 5:** Percentage of piglets with affected body parts and with SINS in suckling piglets and weaners (descriptive data).

	Suckling Piglets	Weaners
Tail base	16.64	21.81
Tail tip	1.81	1.17
Ears	2.8	8.39
Teats	12.85	0.17
Coronary bands	61.78	0
Heels	17.63	0
SINS	72.14	29.1

**Table 6:** Body part scores and effects of farm and sex.

			Farm Coop (Sow and Nursery)			Sex		
		Total	F1 (*n* = 285)	F2 (*n* = 280)	*p*	Male (*n* = 283)	Female (*n* = 282)	*p*
Suckling piglets	Tail base	0.12 ± 0.07	0.23 ± 0.07	0.08 ± 0.10	n.s.	0.11 ± 0.07	0.13 ± 0.07	n.s.
	Tail tip	0.04 ± 0.03	0.02 ± 0.03	0.06 ± 0.04	n.s.	0.05 ± 0.03	0.03 ± 0.03	n.s.
	Ear	0.05 ± 0.02	0.04 ± 0.02	0.05 ± 0.03	n.s.	0.05 ± 0.02	0.05 ± 0.02	n.s.
	Coronary bands	0.63 ± 0.08	0.81 ± 0.07 a	0.54 ± 0.11 b	0.04	0.64 ± 0.08	0.61 ± 0.08	n.s.
	Heels	0.23 ± 0.05	0.16 ± 0.05	0.28 ± 0.08	n.s.	0.23 ± 0.06	0.24 ± 0.06	n.s.
	Teats	0.09 ± 0.07	0.19 ± 0.07	0.04 ± 0.10	n.s.	0.03 ± 0.07	0.14 ± 0.07	<0.001
Weaners	Tail base	0.24 ± 0.07	0.39 ± 0.06	0.19 ± 0.10	n.s.	0.24 ± 0.07	0.24 ± 0.07	n.s.
	Tail tip	0.02 ± 0.01	0.01 ± 0.01	0.02 ± 0.02	n.s.	0.02 ± 0.02	0.02 ± 0.02	n.s.
	Ear	0.10 ± 0.04	0.01 ± 0.04 a	0.14 ± 0.06 ab	n.s.	0.08 ± 0.04	0.13 ± 0.04	0.015
	Coronary bands	Not detected						
	Heels	Not detected						
	Teats	0 ± 0	0 ± 0	0 ± 0.01	n.s.	0 ± 0	0.01 ± 0	n.s.

n.s.: not significant; values with different letters are statistically significantly different (*p* < 0.05).

**Table 7:** Condition of tails at the beginning of fattening phase (all data in % of pigs).

		Farm Coop (Farrowing and Nursery/Finisher)			
	Total	1/1 (*n* = 289)	2/2 (*n* = 192)	2/3 (*n* = 96)	*p*
Tail long intact	44.8 ± 2.5	49.4 ± 3.2 a	39.3 ± 4.1 b	41.4 ± 5.3 ab	n.s.
Tail shorter, no lesions	7.6 ± 1.4	3.3 ± 1.2 a	7.6 ± 2.2 a	19.5 ± 4.3 b	<0.001
Tail shorter, healed lesions	7.6 ± 1.3	4.1 ± 1.3 a	9.0 ± 2.4 ab	14.9 ± 3.8 b	0.006.
Tail longer, acute lesions	29.0 ± 2.4	34.0 ± 1.2 a	29.4 ± 3.8 a	14.9 ± 3.8 b	0.005
Tail short, acute lesions	11.0 ± 1.6	9.1 ± 3.2	15.2 ± 3.0	9.2 ± 3.1	n.s.

n.s.: not significant; values with different letters are statistically significantly different (*p* < 0.05).

**Table 8:** Issues of the tail at slaughter (all data in % of pigs).

		Farm Coop (Farrowing and Nursery/Finisher)			
	Total	1/1 (*n* = 208)	2/2 (*n* = 61)	2/3 (*n* = 94)	*p*
No issue	25.89 ± 6.48	56.37 ± 7.34 a	7.21 ± 4.47 b	29.8 ± 9.77 c	<0.001
Kinks	5.45 ± 2.89	7.58 ± 3.77	4.19 ± 3.57	5.07 ± 3.85	n.s.
Size deviation	6.54 ± 4.06	5.62 ± 3.19	5.15 ± 5.63	9.58 ± 9.07	n.s.
Healed scars	72.83 ± 7.13	39.37 ± 7.65 a	92.68 ± 4.68 b	70.07 ± 10.47 c	<0.001
Ring formation	4.24 ± 2.61	3.14 ± 1.85	6.76 ± 6.1	3.57 ± 3.28	n.s.
Wounds	3.65 ± 2.6	3.69 ± 2.45	3.59 ± 4.49	3.69 ± 4.15	n.s.
Infection	3.31 ± 2.42	3.32 ± 2.27	3.31 ± 4.18	3.3 ± 3.77	n.s.
Necrosis	3.31 ± 2.42	3.32 ± 2.27	3.31 ± 4.18	3.3 ± 3.77	n.s.

Values with different letters differ statistically significantly (*p* ≤ 0.05); n.s.: not significant.

**Table 9:** Tail length of the pigs at slaughter by farm coop.

		Farm Coop (Farrowing and Nursery/Finisher)			
	Total	1/1 (*n* = 208)	2/2 (*n* = 61)	2/3 (*n* = 94)	*p*
>30 cm (*n* = 112)	10.86 ± 4.79	37.46 ± 9.01 a	1.85 ± 2.06 b	13.79 ± 6.78 b	<0.001
23–30 cm (*n* = 141)	35.51 ± 6.37	41.44 ± 6.27 ab	21.48 ± 8.36 a	46.31 ± 10.3 b	0.005
16–22 cm (*n* = 126)	36.79 ± 7.17	16.78 ± 4.47 b	62.64 ± 11.69 a	35.5 ± 10.49 b	<0.001
6–15 cm (*n* = 12)	5.91 ± 3.84	3.5 ± 2.15	11.63 ± 11.15	4.93 ± 5.19	n.s.
<6 cm (*n* = 2)	1.96 ± 1.54	2.49 ± 1.81	2.2 ± 2.7	missing	n.s.

Values with different letters differ statistically significantly (*p* ≤ 0.05); n.s.: not significant.

**Table 10:** Associations between tails at the beginning of fattening and at slaughter. All data are given as percent of slaughter pigs from coop 1.

Tail Status	Tail Status as Fattener						
At Slaughter	Average *n* = 241	Long Intact *n* = 119	Short Intact *n* = 8	Short Healed *n* = 10	Long Acute *n* = 82	Short Acute *n* = 22	*p*
Tail >23 cm	68.6 ± 5.8	95.2 ± 2.1 a ^1^	57.1 ± 18.7 bc	37.5 ± 17.1 b	86.2 ± 4.3 c	33.3 ± 13.6 b	<0.001
Tail >30 cm	0.4 ± 0.3	69.5 ± 4.5 a	0.0 ± 0.0 bc	12.5 ± 11.7 bc	26.2 ± 5.5 c	8.3 ± 8.0 b	<0.001
Tail 16–23 cm	27.7 ± 5.5	3.8 ± 1.9 a	42.9 ± 18.7 bc	50 ± 17.7 bc	12.3 ± 4.1 c	66.7 ± 13.6 b	<0.001
Tail 6–15 cm	0 ± 0.3	0 ± 0	0 ± 0	12.5 ± 11.7	3.1 ± 2.1	0 ± 0	n.s
Tail < 6 cm	0 ± 0	1 ± 0.9	0 ± 0	0 ± 0	0 ± 0	0 ± 0	n.s
No issue	46.5 ± 7.8	76.2 ± 4.2 a	71.4 ± 17.1 a	62.5 ± 17.1 a	29.2 ± 5.6 b	8.3 ± 8.0 b	<0.001
Healed scars	50 ± 7.9	15.2 ± 3.5 a	28.6 ± 17.1 a	37.5 ± 17.1 a	67.7 ± 5.8 b	91.7 ± 8 b	<0.001
Kinks	0.1 ± 0.9	4.8 ± 2.1	0 ± 0	12.5 ± 11.7	6.2 ± 3	8.3 ± 8	n.s
Size deviation	0 ± 0.4	5.7 ± 2.3 a	0 ± 0 b	0 ± 0 b	4.6 ± 2.6	0 ± 0 b	0.050
Ringtail	0.1 ± 0.6	1 ± 0.9	14.3 ± 13.2	25 ± 15.3	3.1 ± 2.1	0 ± 0	n.s
wounds	0 ± 0	1 ± 0.9	0 ± 0	0 ± 0	1.5 ± 1.5	0 ± 0	n.s
infection	0 ± 0	0 ± 0	0 ± 0	0 ± 0	1.5 ± 1.5	0 ± 0	n.s
Necrosis	0 ± 0	0 ± 0	0 ± 0	0 ± 0	1.5 ± 1.5	0 ± 0	n.s

Percentage of slaughter pigs with tail characteristics in association to their status at the beginning of fatteners. Data are calculated with a general linear model for binary data with the tail status at the beginning of fattening as fixed effect and the tail characteristics at slaughter as dependent variable. Data are given as mean percentage of pigs with the slaughter characteristic ± standard error. *p*: significance; n.s.: not significant; ^1^: values with the same letter are not statistically significant (*p* ≤ 0.05).

**Table 11:** Associations between tails length and tail acuteness at the beginning of fattening and at slaughter. All data are given as percent of slaughter pigs from coop 1.

	Tail Status at Fattening					
Tail at Slaughter	Shorter	Longer		Not Acute	Acute	
Tail >23 cm	38.9 ± 9.6	91.6 ± 2.2	<0.001	81.0 ± 5.1	62.0 ± 8.1	0.04
>30 cm	5.1 ± 3.7	47.6 ± 4.4	<0.001	2.5 ± 1.9	8.9 ± 3.4	<0.001
23–30 cm	33.5 ± 9.5	42.1 ± 4	n.s.	39.1 ± 6.9	26.4 ± 4.7	<0.001
16–22 cm	57.5 ± 9.9	7.1 ± 2	<0.001	49.8 ± 7	56.8 ± 5.3	0.029
6–15 cm	3.4 ± 3.5	1.2 ± 0.8	n.s.	8 ± 3.9	4.2 ± 2.1	n.s.
<6 cm	0 ± 0	0 ± 0.6	n.s.	0 ± 0	0 ± 1.1	n.s.
No issues	34.8 ± 10.1	53.2 ± 4.5	<0.001	18.2 ± 5.4	16 ± 4	n.s.
Kinks	7.5 ± 5.1	5.4 ± 1.8	n.s.	11.9 ± 4.6	4.5 ± 2.2	n.s.
Local size deviation	0 ± 0	5.1 ± 1.8	0.004	9.4 ± 4.1	3.4 ± 1.9	0.004
Healed scars	67 ± 10.3	38 ± 4.5	0.01	24.0 ±5.2	79.8 ± 5.3	<0.001
Ringtail	10.8 ± 6	1.7 ± 1	0.021	4 ± 2.8	3.2 ± 1.9	n.s.
Open wound	0 ± 0	0 ± 0	n.s.	0 ± 0	0 ± 0	n.s.
infection	0 ± 0	0 ± 0	n.s.	0 ± 0	0 ± 0	n.s.
Necrosis	0 ± 0	0 ± 0	n.s.	0 ± 0	0 ± 0	n.s.

n.s.: not significant.

## Data Availability

Data is unavailable due to privacy restrictions, but any data used or analyzed during the current study is available from the authors on reasonable request.

## Supplementary Materials

The following supporting information can be downloaded at: https://www.mdpi.com/article/10.3390/ani16010056/s1, Table S1. Body part scores by age of suckling piglets; Table S2. Body part scores by parity.
