# Supplementary material for: Longitudinal Study: Swine Inflammation and Necrosis Syndrome in Suckling and Weaned Piglets Is Associated with Tail Length and Integrity in Slaughter Pigs
Source: Animals (Basel). 2025 Dec 24;16(1):56. doi: 10.3390/ani16010056 (PMC12785067; doi:10.3390/ani16010056)
Supplement: Supplementary file 1 [file animals-16-00056-s001.zip › animals-4050587-supplementary.pdf]

Supplementary Materials

Longitudinal Study: Swine Inflammation and Necrosis Syndrome in Suckling and Weaned Piglets is Associated with Tail Length and Integrity in Slaughter Pigs

Karien Koenders-van Gog [1], Thomas Wijnands [1], Mirjam Lechner [2] and Gerald Reiner [3,*]

[1] Lintjeshof Veterinary Practice, LH Vet Group, 6031 RK Nederweert, The Netherlands
[2] UEG Hohenlohe, 91567 Herrieden, Germany
[3] Clinic for Swine—Herd Health Management and Molecular Diagnostics, Justus-Liebig-University Giessen, 35392 Giessen, Germany
* Correspondence: gerald.reiner@vetmed.uni-giessen.de; Tel.: +49-641-99-38821

Supplemental Tables

Table S1. Body part scores by age of suckling piglets.

		**Age in days**							
		**0 (n=23)**	**1 (n=284)**	**2 (n=39(**	**3 (n=14)**	**4 (n=34)**	**5 (n=55)**	**6 (n=116)**	**P**
Suckling piglets	Tail base	0.56 ± 0.18ac	0.31 ± 0.07c	0.47 ± 0.15c	-0.13 ± 0.24bc	-0.14 ± 0.16b	-0.11 ± 0.17b	-0.12 ± 0.13b	0.058
	Tail tip	0.11 ± 0.07	0.06 ± 0.03	0.03 ± 0.06	0 ± 0.09	0.03 ± 0.06	-0.01 ± 0.07	0.05 ± 0.05	n.s.
	Ear	0.05 ± 0.05	0 ± 0.02	0 ± 0.04	0.07 ± 0.07	0.04 ± 0.04	0.1 ± 0.05	0.07 ± 0.04	n.s.
	Coronary bands	0.78 ± 0.19	0.73 ± 0.08	0.81 ± 0.16	0.61 ± 0.25	0.57 ± 0.17	0.31 ± 0.18	0.57 ± 0.14	n.s.
	Heels	0.53 ± 0.13a	0.42 ± 0.05a	0.55 ± 0.11a	0 ± 0.18b	0.1 ± 0.12b	0.04 ± 0.12b	0 ± 0.1b	0.037
	Teats	0.09 ± 0.18	0.16 ± 0.07	0.12 ± 0.16	0 ± 0.25	0.01 ± 0.16	0.17 ± 0.17	0.04 ± 0.13	n.s.

n.s.: not significant; values with different letters are statistically significantly different (P < 0.05).

Table S2. Body part scores by parity.

		Parity<br>1 (n=213)	2 (n=159)	3 (n=83)	4 (n=40)	5 (n=70)	P
Suckling piglets	Tail base	-0.05 ± 0.12a	0.14 ± 0.11ab	0.25 ± 0.13b	0.11 ± 0.14ab	0.16 ± 0.11ab	n.s.
	Tail tip	-0.01 ± 0.05a	0.06 ± 0.04a	-0.03 ± 0.05a	0.17 ± 0.05b	0 ± 0.04a	0.041
	Ear	0.05 ± 0.03a	-0.02 ± 0.03a	0.12 ± 0.03b	0.05 ± 0.04ab	0.03 ± 0.03a	0.023
	Coronary bands	0.33 ± 0.13a	0.7 ± 0.11b	0.61 ± 0.13b	0.85 ± 0.14b	0.64 ± 0.11b	0.01
	Heels	-0.1 ± 0.09c	0.31 ± 0.08ab	0.37 ± 0.09a	0.44 ± 0.1a	0.16 ± 0.08b	<0.001
	Teats	0.04 ± 0.12	0.04 ± 0.11	0.07 ± 0.13	0.06 ± 0.14	0.23 ± 0.11	n.s.
Weaners	Tail base	0.12 ± 0.11a	0.17 ± 0.09ab	0.45 ± 0.11b	0.13 ± 0.12ac	0.35 ± 0.1ac	0.005
	Tail tip	0.02 ± 0.02ac	0 ± 0.02ac	0.06 ± 0.02b	0 ± 0.03ac	0 ± 0.02c	0.12
	Ear	0.09 ± 0.07	0.12 ± 0.06	0.07 ± 0.07	0.1 ± 0.07	0.14 ± 0.06	n.s.
	Coronary bands	Not detected					
	Heels	Not detected					
	Teats	0.01 ± 0.01ac	-0.01 ± 0.01b	0.01 ± 0.01c	-0.01 ± 0.01b	0.02 ± 0.01ac	0.004

n.s.: not significant; values with different letters are statistically significantly different ($P < 0.05$).
